# Using default opt-out strategies to undercover the unknown HCV infections: a scoping review

**DOI:** 10.1093/eurpub/ckac130.233

**Published:** 2022-10-25

**Authors:** L Ceccarelli, G Paparatto, L Tavoschi

**Affiliations:** 1 Department of Translational Research in Medicine, University of Pisa, Pisa, Italy; 2 Management and Healthcare Laboratory, Sant’Anna School of Advanced Studies, Pisa, Italy

## Abstract

**Background:**

Following the advent of directly acting antivirals (DAAs) a global effort is underway to eliminate viral hepatitis C (HCV) by 2030. Yet identification of infected individuals and access to dedicated services may pose a challenge to the achievement elimination targets. A scoping review to synthesize studies that explored the efficacy of opt-out strategies to improve HCV testing capacity was conducted.

**Methods:**

We searched PubMed and Scopus (from 2015 to March 2022) for all English original articles and systematic reviews addressing opt-out strategies for HCV testing in different settings, published in the WHO's European Region Countries. We excluded articles that focused on other testing implementation strategies.

**Results:**

A total of 136 articles were screened at the title and abstract level, of which 41 were also assessed at full text for eligibility after deduplication. In the end, 30 articles met the inclusion criteria. Studies originated from 19 different countries of the WHO's European Region, with the most prevalent being France (26.9%, 11/41). The 43.3% of the articles addressed opt-out testing strategies in emergency departments (EDs), 36.6% into prisons, 13.3% in primary care, and 6.6% among people who use drugs. Opt-out default testing was found to be effective in EDs and prisons, whereas only 2 articles tested the efficacy of opt-out strategies for HCV testing in primary care settings.

**Conclusions:**

Opt-out strategies resulted in increased testing rates and higher cost-effectiveness in different settings, especially EDs and prisons. However, to identify individuals with undiagnosed infections, birth cohorts screening in the general population may be needed. Further research is needed to assess the utility of an opt-out default testing strategy in primary care settings.

**Key messages:**

- Opt-out strategies resulted in increased testing rates and higher cost-effectiveness in different settings.
- OpT-out strategies should be implemented in primary care settings to undercover the unknown infections.

